# Magnitude of Malaria and Factors among Febrile Cases in Low Transmission Areas of Hadiya Zone, Ethiopia: A Facility Based Cross Sectional Study

**DOI:** 10.1371/journal.pone.0154277

**Published:** 2016-05-03

**Authors:** Romedan Kedir Delil, Temesgen Kale Dileba, Yitagesu Aweke Habtu, Terefe Fuge Gone, Taye Janfa Leta

**Affiliations:** 1 Department of Clinical Nursing, Hossana College of Health Sciences, Hossana, Ethiopia; 2 Department of Health Informatics, Hossana College of Health Sciences, Hossana, Ethiopia; 3 Department of Medical Laboratory Sciences, Hossana College of Health Sciences, Hossana, Ethiopia; 4 Department of Public Health, Hossana College of Health Sciences, Hossana, Ethiopia; Centro de Pesquisa Rene Rachou/Fundação Oswaldo Cruz (Fiocruz-Minas), BRAZIL

## Abstract

**Background:**

Despite a remarkable decline in morbidity and mortality since the era of malaria roll back strategy, it still poses a huge challenge in Ethiopia in general and in Hadiya Zone in particular. Although, there are data from routine health management information on few indicators, there is scarcity of data showing magnitude of malaria and associated factors including knowledge and practice in the study area. Therefore, the aim of this study was to assess magnitude and factors affecting malaria in low transmission areas among febrile cases attending public health facilities in Hadiya Zone, Ethiopia.

**Methods:**

A facility based cross-sectional study was conducted in Hadiya Zone from May 15 to June 15, 2014. Simple random sampling was used to select the health facility while systematic random sampling technique was used to reach febrile patients attending public health facilities. Data were collected by a pre-tested structured questionnaire containing sections of socio demographic risk factors and knowledge and prevention practices of malaria. Data were entered to Epi-Info software version 3.5.4 and exported to SPSS version 16 for descriptive and logistic regression analysis.

**Results:**

One hundred six (25.8%) of participating febrile patients attending at sampled health facilities were found to have malaria by microscopy. Of which, *P*.*vivax*, *P*.*falciparum* and mixed infection accounted for 76(71. 7%), 27 (25.5%) and 3 (2.8%), respectively. History of travel to malaria endemic area, [AOR: 2.59, 95% CI: (1.24, 5.38)], not using bed net, [AOR: 4.67, 95%CI:, (2.11, 10.37)], poor practice related to malaria prevention and control, [AOR: 2.28, (95%CI: (1.10, 4.74)], poor knowledge about malaria, [AOR: 5.09,95%CI: (2.26,11.50)] and estimated distance of stagnant water near to the residence, [AOR: 3.32, (95%CI: (1.13, 9.76)] were significantly associated factors of malaria positivity in the study.

**Conclusion:**

The present study revealed that malaria is still a major source of morbidity in the study area among febrile illnesses. Poor level of knowledge, poor prevention practices, not using bed net, travel history to endemic areas and residing near stagnant water were associated factors with malaria positivity in the study area. Therefore, implementers, policy makers and stakeholders should strengthen the services provided by the community health development army, health extension service and health facilities services focusing on increasing malaria intervention coverage and mobilization of information, education and communication to increase knowledge about malaria transmission, prevention and control practices.

## Background

Globally, an estimated 3.3 billion people are at risk of being infected with malaria and 1.2 billion are at high risk (>1 in 1000 chance of getting malaria in a year). According to the 2014 WHO report, 198 million cases of malaria occurred globally (uncertainty range 124–283 million) and the disease led to 584 000 deaths (uncertainty range 367 000–755 000)[1]. Although facts showed a decline of malaria burden, decreases in case incidence and mortality rates were slowest in countries that had the largest numbers of malaria cases and deaths in 2000. The global burden of mortality is dominated by countries in sub-Saharan Africa, where an estimated 90% of all malaria deaths occur, and in children aged under 5 years, who account for 78% of all deaths [1].

Despite a remarkable decline in morbidity and mortality due to malaria as of malaria roll back era, it still poses huge challenge. Malaria is ranked as the leading communicable disease in Ethiopia, accounting for about 30% of the overall Disability Adjusted Life Years lost. **[2]**. According to records from the Ethiopian Federal Ministry of Health, 75% of the country is malarious with about 68% of the total population living in areas at risk of malaria. That is, more than 50 million people are at risk from malaria [2,3], and four to five million people are affected by malaria annually, of these, *P*.*falciparum* and *P*. *vivax* accounts for 60% and 40% of cases respectively. However, the malaria species distribution varies with spatial geography [4, 5] and the risk has not recently been revised considering possible changes such as urbanization and land use [5].

The Federal Ministry of Health (FMoH) of Ethiopia plans a strategy to achieve malaria elimination within specific geographical areas with historically low malaria transmission and zero deaths due to malaria in areas with malaria transmission by 2020 [6]. Because there were gaps in the surveillance system, official estimates of the true burden of malaria in Ethiopia needs supplementary evidences with regard to malaria magnitude by species. Moreover, there is no report on active case detection of reactive and febrile cases at community level and mass screening in intervention policies and strategies in the country **[1].** The health management information system of Ethiopia enables only to estimate from both laboratory and clinically confirmed malaria morbidity reports [7]. One epidemiological thematic review conclude that in addition to the magnitude of disease, the distribution species of malaria has to be given equal weight for the prevention, control and elimination of malaria [2].

In Ethiopia, coexistence of both *P*. *falciparum* and *P*. *vivax* can create challenge in prevention, control and elimination of the disease [8]. The current focus of control in Africa including Ethiopia is justifiably on P. falciparum, by far the most pathogenic of the five human malarias, contributing to over 95% of the world’s mortality from malaria [8]. However, far less is known about the epidemiological distribution or clinical consequences of *P*.*vivax* [2].

In the previous couple of years, malaria indicators surveys have been conducted and surveillance and facility based reports used to describe the burden of malaria in Ethiopia. However, facility based studies indicated that there are significant gaps in continuous laboratory supplies and reagents, and lack of training and supportive supervision [9]. Another study conducted in Kenya showed more than half of the febrile cases were wrongly diagnosed as if they were truly clinical malaria [10]. Continuous research evidences are needed through systematic scientific studies for malaria control, prevention and elimination interventions. Moreover, in Ethiopia where there is no active case detection of reactive and febrile cases at community level, a facility based study has indispensable support for area and context specific intervention [8].

Despite impressive gains in malaria intervention coverage for sub-Saharan Africa, millions of people still do not receive the services they need. Lack of access to an ITN or IRS, gaps in providing universal access to diagnostic testing and treatment, decrease in health care service utilization due to weaknesses in health systems(decrease in health care seeking), resistance to insecticides used in ITNs and IRS, anti-malarial drug resistance [1] remains the principal barriers to protection from mosquito bites.

Ethiopia is a very ecologically diverse country and harbors many different epidemiological profiles for malaria transmission. In epidemic-prone areas there is significant heterogeneity in transmission risk, and as a result, elimination strategies will not be uniform throughout the country. Each area may require its own unique interventions, which in turn would be delivered progressively until elimination can be achieved [6]. Due to these challenge, this study tried to identify the magnitude of malaria species among febrile cases attending public health facilities to support the existing malaria prevention, control and elimination strategies of the area.

Treatment seeking behavior is also important for early case detection and management so that transmission would be reduced. In Ethiopia, the national Malaria Indicator Survey (MIS) undertaken in 2011 showed that over 40% of fevers were treated within 24/48 hours with an anti-malarial drugs outside the formal public health reporting system [11]. These features of malaria treatment pose challenges to case-management but equally to the reliability of active case detection completeness [8]

Furthermore, attention of malaria burden in low transmission settings is lower which may pave the way for vector control and integrated vector management [12]. Despite this, the preceding malaria researches in Ethiopia majorly focused on endemic transmission areas and as a result, there have been a scarcity of studies addressing magnitude, distribution of species and factors in low transmission areas [13–14].

Although, there are data from routine health management information on few indicators, there is a need for area and context specific data showing magnitude of malaria and associated factors including knowledge and practice essential to achieve Ethiopia’s strategic plan [6]. Several factors including ownership and utilization of LLITNS, knowledge and practice of the community, and socio-economic, treatment seeking behavior, socio-demographic, environmental factors need to be assessed with regard to malaria status. Studies revealed that knowledge and practices about malaria, household location for stagnant water sources, bed net utilization, age, and travel history, are some of the factors associated with malaria [11, 15, 16, 17, 18].

Therefore, there is a need of area and context specific evidences about malaria magnitude, species distribution and factors with high susceptible cases both rural and urban area of low transmission settings including highland areas. This in turn enhances design and implementation of cost-effective appropriate intervention methods to realize the elimination strategy set by the country. Hence, the aim of this study was to determine the magnitude of malaria with its species distribution and factors associated with malaria status among febrile cases in low transmission areas of Hadiya zone in Ethiopia. The finding will contribute for supplementary information for policy makers and implementers as well as scientific society to support malaria control and elimination strategy.

## Methods

### Study area

This study was conducted in governmental health facilities found in Hadiya Zone, Southern Ethiopia. The study area is located in 7^0^ 30’ (75^0^) north latitude and 37^0^ 45’ (37.75^0^) east longitude with an average elevation of 2,106 meters above sea level. According to the geo malaria category of the country, the study area is under the classification of low transmission areas with expected sporadic epidemics every few years [4]. Eighty nine percent of the population lives in rural area and only 10.7% live in urban areas. The zone has one zonal hospital, three primary hospitals under construction and three hundred health post each expected to serve average 1 million people, 60,000 to 100,000 people, 15,000 to 25,000 and 3,000 to 5,000 people, respectively. According to the report of the zonal health bureau, malaria is one of the leading causes of morbidity among outpatient and inpatient departments of all health facilities.

### Study Design

Facility based Cross-Sectional study was applied from May to June, 2014.

### Sample Size and Sampling techniques

The minimum sample size for the study was estimated by using single population proportion formula [19] taking 95% confidence level (CI), (Z (1-ά/2) = 1.96), 50% expected proportion of slide positivity (p = 0.5) to maximize the sample size because there was no previous proportion of slide positivity near the study area, 5% margin of error (d = 0.05). Using the above assumptions, the sample size was calculated as follows.

$$n=\frac{{\left(Z1-\frac{\alpha }{2}\right)}^{2}\;P(1-P)}{d^{2}}$$

$$n=\frac{{\left(1.96\;\right)}^{2}\;0.5(1-0.5)}{{\left(0.05\right)}^{2}}=384$$

and 10% (38) non-response rate, a minimum sample of 422 participants were needed.

The study was conducted in twelve health centers accounting 20% of all health centers (60 health centers) found in the Zone. Health facilities were selected using simple random sampling by assuming they are nearly similar in terms of malaria burden, catchment population demographic risk factors, and access to malaria prevention to get representative participants. A systematic random sampling technique was used to reach febrile patients attending in selected health centers to get a representative understanding of malaria knowledge, practice and related factors among the febrile cases of population in Hadiya Zone.

### Data Collection

Twelve clinical/public health officers and twelve medical laboratory technicians and 2 supervisors were trained for three days to maintain quality of data prior to the actual data collection period.

During data collection, the prior five days average number of febrile cases were taken then multiplied by the data collection period to determine the average total number of febrile cases. Then, the total value was divided by the sample size allocated for each health facility to determine K^th^ values and 1 up to K element were selected by lottery method. Patients were first clinically diagnosed to rule out history of fever according to diagnosis protocol by trained clinical nurses/health officer’s staff existing in selected health facilities [20]. Those participants eligible to be included were selected consecutively based on a systematic interval. Febrile adults whose age was greater than 18 years and care givers of those less than 18 years were interviewed about socio-demographic, risk factors including knowledge and practices of malaria.

After the interview was complete, blood slide samples were taken and examined by 12 trained laboratory technologists of existing staff at health facilities. Then, blood slides were diagnosed for malaria parasite by direct microscopy techniques according to National Malaria diagnosis Guideline (NMG) of malaria guideline i.e. blood samples were obtained through finger prick method for thick and thin smears [20]. The blood smears were prepared on microscope slides and stained using 10% Giemsa to be examined under 100x microscopes for the presence of malaria parasites [21]. If one or more asexual stage of plasmodium (trophozoite, ring stage, merozoite and/or gametocyte) is/are present, it was taken as a positive result. Five percent of the slides were randomly selected and then blindly checked for consistency by experienced medical laboratory technologist.

### Operational Definitions

#### Poor Knowledge

When a knowledge score is equal to or less than first quartile of the total score of 15 item questions on areas of malaria prevention, control, transmission and health seeking behaviors.

#### Medium Knowledge

When a knowledge score is between the first and second quartile of the total score of 15 item questions on areas of malaria prevention, control, transmission and health seeking behaviors.

#### Good Knowledge

When a knowledge score is greater than third quartile of the total score of 15 item questions on areas of malaria prevention, control, transmission and health seeking behaviors.

#### Poor Practice

When a reported practice score is less or equal to first quartile of the total score of 7 item questions on areas of malaria prevention, control, transmission and health seeking behaviors.

#### Good Practice

When a reported practices score is greater than third quartile of the total score of 7 item questions on areas of malaria prevention, control, transmission and health seeking behaviors.

#### Parasite density

If 1–10 parasites per 100 thick high power fields, it was taken as +1, 11–100 parasites per 100 high power fields +2, 1–10 parasites in every high power field +3 and more than 10 in every high power field +4 [20]

### Ethical Consideration

Ethical clearance was obtained from Research Ethical Review Committee of Hossana College of Health Sciences. Information about the objectives of the study, confidentiality, autonomy and justice was explained to the participants during data collection. Written consent was also obtained from each study participant based on the approval by the institutional review board of the college. All individuals who had fever on physical examination and were positive for malaria parasites during blood film examination were offered anti-malarial treatment as per national guidelines [21].

### Data Processing and Analysis

The collected data were cleaned and checked for consistencies and completeness then entered use EPI info version 3.5.4 and exported to SPSS 16. Both descriptive and logistic regression was used to identify factors. Standard multiple regression was used to identify how much variance in test positivity status the factors were able to explain as a group or block [22]. All variables which showed statistically significant results with malaria status in binary logistic regression were entered to multivariate logistic regression to control for confounders.

## Result

### Socio-demographic characteristics

A total 411 of 422 participants were involved in the survey making the response rate of 97.4%. Three hundred sixty six (89%) were febrile adults while the rest were caregivers of febrile children under 18. The age of the respondents ranged from 18 years to 70 years with a mean age of 30.7 years. Almost equal number of males (50.4%) and females (49.6%) were involved in the study giving a male to female ratio of 1:1. The average monthly income of the study population ranged from 4.8$ to 390.5$ with mean income of 54$ US dollar (Table 1).

**Table 1:** Socio-demographic characteristics among attendants of public health facilities in Hadiya Zone, Southern Ethiopia, 2014 (n = 411).

Socio-demographic characteristics	Frequency	Percent
Sex of respondents		
Female	204	49.6
Male	207	50.4
Age category		
18–24	117	28.5
25–40	234	56.9
41–60	55	12.4
≥60	5	1.2
Head of household		
Male	365	88.8
Female	46	11.2
Marital status		
Married	291	70.8
Single	104	25.3
Divorced	7	1.7
Widowed	5	1.2
Separated	4	1.0
Ethnicity		
Hadiya	361	87.8
Kambata	28	6.9
Wolayta	3	0.7
Oromo	3	0.7
Others	16	3.9
Educational status		
Unable read and write	59	14.4
Grade 1–4	69	16.8
Grade 5–8	116	28.2
Grade 9–10	89	21.7
Grade 11–12	34	8.3
College and above	44	10.7
Estimated monthly income in USD		
≤57$	276	67.2
57$-76$	48	11.7
76$-114$	44	10.7
≥114$	43	10.4
Construction material for wall of a house		
Thatch	178	43.3
Bricks	201	48.9
Burnt brick with cement	18	4.4
Other	14	3.4
Family Size		
1–4	126	30.7
5–8	235	57.2
≥8	50	12.2

### Malaria and related factors

One hundred and six respondents had malaria which gave a slide positivity of 25.8% in the study area. Out of the 106 cases, 76 (71.7%), 27 (25.5%), 3 (2.8%) were *P*.*vivax*, *P*.*falciparum* and were mixed infections respectively. Microscopic examination also revealed the parasite load of the reactive cases. Accordingly, 72(67.9%) of the cases were low (+1) and the remaining 10.4% and 21.7% of the cases carried moderate (+2) and high (+3 or +4) parasites load, respectively (See Table 2).

**Table 2:** Malaria and related factors among attendants of public health facilities in Hadiya Zone, Southern Ethiopia, 2014 (n = 411).

Malaria and related factors	Frequency	Percent
Blood film result		
Positive	106	25.8
Negative	305	74.2
Species of malaria identified		
*P*. *vivax*	76	71.7
*P*.*falciparum*	27	25.5
Mixed infection	3	2.8
Blood film parasite load		
+1	72	67.9
+2	11	10.4
+3	3	2.8
+4	20	18.9
Distance of residence from surface water**		
≤500 m	143	34.8
500-1500m	154	37.5
1501–3000 m	43	10.4
≥3000 m	71	17.3
Travel history to endemic area***		
Yes	75	18.3
No	336	81.7
Had home visit by HEW		
Yes	326	79.3
No	85	20.7
Frequency of home visit by HEW		
Daily	2	0.5
Weekly	84	25.9
Monthly	154	47.4
Others (every six month, yearly)	86	26.2
Malaria is transmitted by		
Fly	32	7.8
Any mosquito	194	47.2
Bite of plasmodium infected *Anopheles* mosquito	109	26.5
Cockroach	4	1.0
I don’t know	72	17.5
Malaria mosquito feed at		
Day time	25	6.1
Night time	209	50.9
Day and night time	77	18.7
I don’t know	100	24.3

** Estimated by one reported trip travel time (1 minute for every 30 meters for educated participants and pointing the available distance during data collection for non-educated and converting to similar units for permanent and temporary stored water sources in the last 30 days.

*** Judged by asking when and where they had travelled in the last 30 days and determine based on risk classification of the Zone

### Knowledge about malaria and practice of its prevention methods

Regarding knowledge of respondents on the way of malaria transmission, only 26.5% of the respondents replied that malaria is transmitted by the bite of *Plasmodium* infected mosquitoes (See Table 2). On the other hand, 80% of the respondents reported that sleeping under bed net prevents malaria transmission from infected person to non-infected person (See Table 3).

**Table 3:** Participants response about malaria related factors among attendants of public health facilities in Hadiya Zone, Southern Ethiopia, 2014 (n = 411).

Malaria related factors	Responses			
	Yes		No	
	Frequency	Percent	Frequency	Percent
**Ever heard about Malaria**	376	91.5	35	8.5
Malaria transmission***				
Drinking contaminated water	76	18.5	335	81.5
Eating contaminated food	29	7.1	382	92.9
Eating a lot of mango	11	2.7	400	97.3
Bite of mosquito infected	302	73.5	109	26.5
Close contact with malaria patient	48	11.7	363	88.3
Ways to prevent and control malaria***				
Sleeping in bed nets	329	80	82	20
Wearing long sleeved clothes	85	20.7	326	79.3
Making fire and smoke	40	9.7	371	90.3
Spraying insecticide	158	38.4	253	61.6
Trimming bushes around house	26	6.3	385	93.7
Cleaning dark corners 0f house	65	15.8	346	84.2
I don’t know	25	6.1	386	93.9
Personal protection measures***				
Use repellents	139	33.8	272	66.2
Use mosquito coil	130	31.6	281	68.4
Use doom	6	1.6	405	98.4
Burn cow dung/leaves	44	10.7	367	89.3
Close window and doors	100	24.3	311	75.7
Gauze wire in the window	57	13.9	354	86.1
Use mosquito net	187	45.5	224	54.5
No practice of protection	27	6.6	384	93.4
Others	10	2.4	401	97.6

*** Analyzed as one participant could respond multiple options or only one option.

Regarding awareness, the majority of participants (91.5%) had ever heard about malaria from different sources of information. Accordingly, the major sources of information were radio transmission and health center/clinic which accounts for 51.3% and 50.4%, respectively.

Only 29.9% % of participants had sought treatment within the first 24 hours of symptom onset while the rest had delayed treatment seeking behavior. Including the recommended time of treatment seeking behavior (24 hours from onset of symptom), participants were also asked about where they go for treatment when they had experienced similar symptoms before. Based on this, 268 (65.2%) of the respondents reported that they had sought treatment from health center/clinic, 119 (29%) of them had sought from drug shop/pharmacy and 80 (20%) of them had sought from community health workers mainly health extension workers at any time with similar febrile illnesses (see Table 4).

**Table 4:** The distribution of source of information and treatment seeking for malaria among respondents in Hadiya zone, Southern Ethiopia, 2014 (n = 411).

Source of information and Treatment seeking behavior	Response			
	Yes		No	
	Frequency	Percent	Frequency	Percent
Source of Information **				
Family members at home	108	26.3	303	73.7
Neighbors in the village	130	31.6	281	68.4
Radio	211	51.3	200	48.7
Television	94	22.9	317	77.1
News paper	12	2.9	399	97.1
Posters or pamphlets	23	5.6	388	94.4
School	105	25.5	306	74.5
Church	24	5.8	387	94.2
Health secretary of Local community	6	1.5	405	98.5
Village health team	83	20.2	328	79.8
Health center/Clinic	207	50.4	204	49.6
Community health workers	189	46	222	54
Other sources	9	2.2	402	97.8
Treatment seeking at 24 hours of onset symptoms	123	29.9	288	70.1
Places of treatment seeking at any time of fever**				
Health center/Clinic	268	65.2	143	34.8
Community health workers	82	20	329	80
Traditional healer	10	2.4	401	97.6
Drug shop/Pharmacy	119	29	292	71
Look for local herbs	12	2.9	99	97.1
I don't know	6	1.5	405	98.5
Not at all previously	10	2.4	401	97.6

** One participant may respond more than one option

### Bed net Ownership, Utilization and other prevention practices

**Fig 1:**
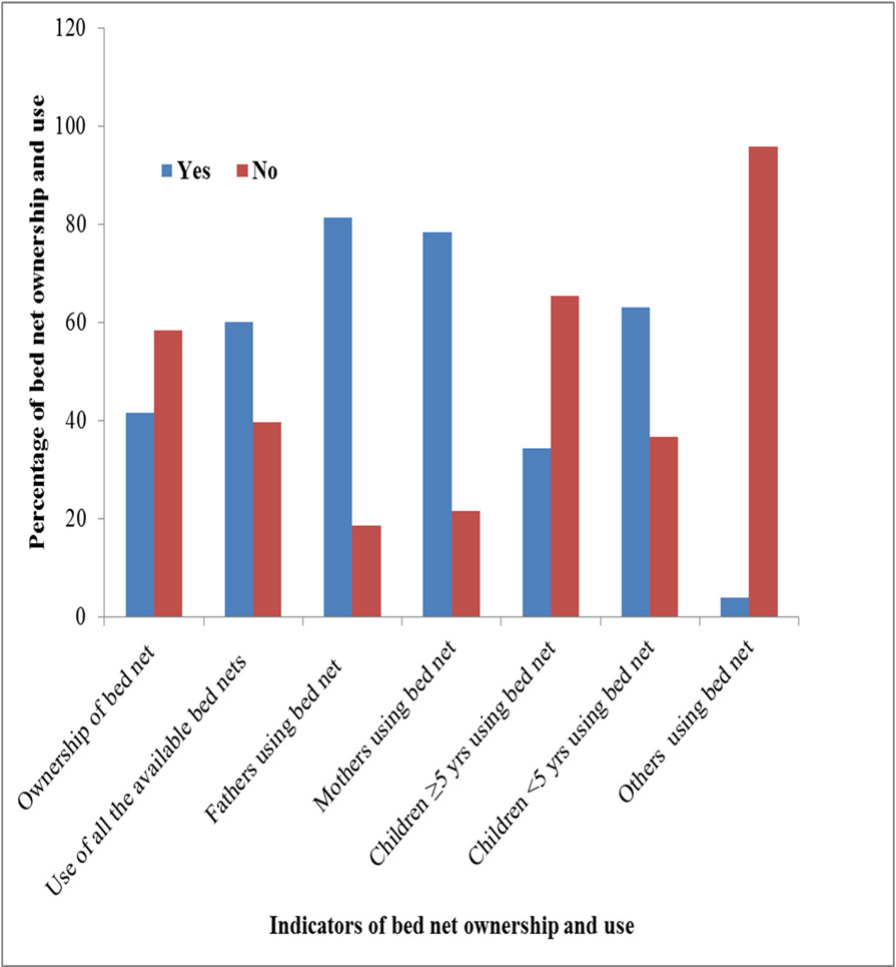
Distribution of bed net ownership and use among attendants of public health facilities in Hadiya Zone, Southern Ethiopia, 2014 (n = 411). Legends: blue color or “Yes” expresses the presence of labeled items. Red color or “No” expresses the presence of labeled items.

Of the respondents, only one hundred and seventy one households had bed net in their home making the bed net ownership proportion 41.6% in the study area. Among the family members found in the study households, 81.3% of the fathers, 78.4% of the mothers, 34.5% of the children over five years of age and 63.2% of the children under five years of age were reported as having a bed net. The study also revealed that more than half of the individuals who owned bed nets (60.2%) reported that they were using it appropriately. The major reasons reported for not using bed net were lack of supply (50.8%), inadequate number to family size (38.5%), oldness of bed nets (7.7%) and participants’ perception of absence of malaria in the area 2 (3.1%) (Fig 1).

**Fig 2:**
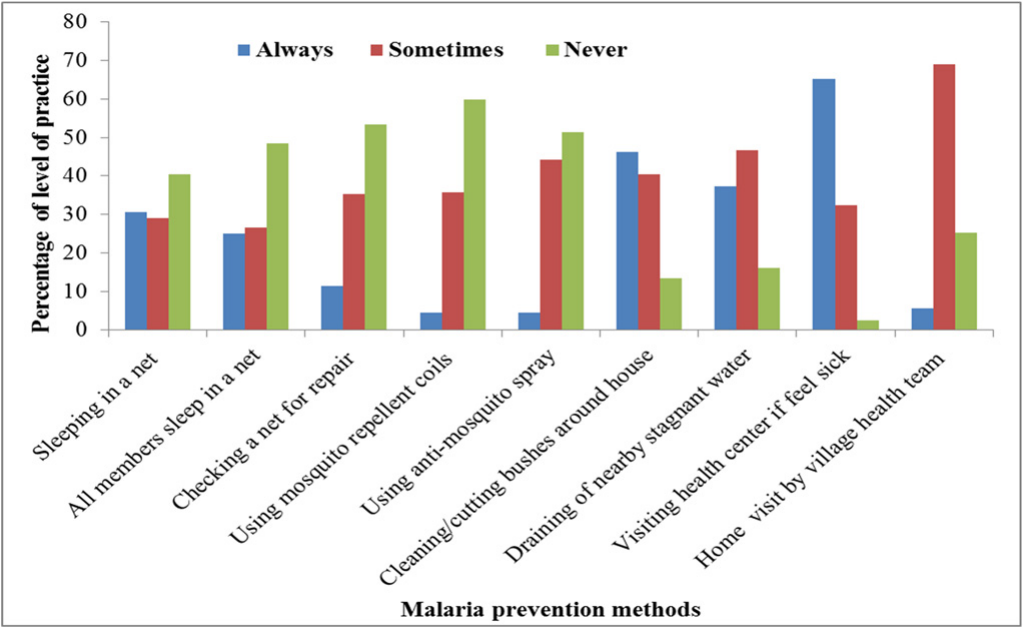
Level of practice on malaria prevention among attendants of health facilities in Hadiya zone, Southern Ethiopia, 2014 (n = 411). Legend: Blue color: expresses “they always practice the specified prevention items”. Red color or: expresses “they sometimes practice the specified prevention items”. Light green color: expresses “they never practice the specified prevention items”.

Participants were also interviewed to provide multiple practices for malaria prevention. One hundred ninety, (46.2%) of the study participants clean/cut bushes around their house and 153 (37.2%) of them drain stagnant water near their home, 200 (48.7%) household spray at every six month, 165 (40.2%) used both traditional mosquito repellents and other methods (Fig 2). More than half of the respondents (53.3%) never practiced repairing of mosquito net to prevent mosquito bite. Similarly, 246 (59.9%) and 211 (51.3%) of the respondents never used mosquito repellent and anti-mosquito spray respectively to prevent mosquito bite (Fig 2).

### Knowledge and reported Practice items score summary

From the summary scores of knowledge and reported practice items, only 77(18.7%) of the respondents had good knowledge on causes, mode of transmission, prevention and control and treatment seeking behavior for malaria while 241 (58.6%) of them had poor reported practice on prevention and control measures and treatment seeking behavior for malaria (See Table 5).

**Table 5:** Knowledge about and reported practice on prevention, control, transmission and treatment seeking behaviors of malaria among febrile attendants of public health facilities in Hadiya Zone, Southern Ethiopia, 2014 (n = 411).

Knowledge and Practice Score	Frequency	Percent
Knowledge Score	** **	** **
Poor Knowledge	136	33.1
Medium Knowledge	198	48.2
Good Knowledge	77	18.7
Total	411	100
Practice Score	** **	** **
Poor Practice	241	58.6
Good Practice	170	41.4
Total	411	100

### Factors associated with malaria positivity

Among the selected variables, travel history, bed net use, knowledge about malaria and practice of prevention methods showed a significant association with malaria positivity when adjusted for all other variables. Individuals with a history of travelling to malaria endemic area were 2.59 times more likely to be malaria positive when compared to those who did not [AOR: 2.59, 95% CI: (1.24, 5.38)]. Similarly, those who were not using bed net were 4.67 times more likely to be infected by *Plasmodium* than bed net users [AOR: 4.67, 95%CI: (2.11, 10.37)]. In the same fashion, participants who were poorly practicing prevention methods were 2.28 times more likely to be malaria positive as compared to those who were practicing good [AOR: 2.28, (95%CI: (1.10, 4.74)]. Likewise, those having poor knowledge about malaria were five folds more likely to have the disease when compared to those who had good knowledge prevention methods [AOR: 5.09, 95%CI: (2.26, 11.50)]. However, medium knowledge score was not associated with malaria positivity [AOR: 0.89, 95% CI: (0.38, 2.07)]. Estimated distance from the respondents residence to stagnant water was not associated with malaria positivity in the multivariate analysis in general but those resided at a distance of 500 to 1500 meter away were 3.32 more likely to be malaria positive when compared to those residing at a distance more than 3000 meters away from stagnant water [AOR: 3.32, (95%CI: (1.13, 9.76)] (See Table 6).

**Table 6:** Factors of malaria among attendants of public health facilities of Hadiya Zone, Southern Ethiopia, 2014 (n = 411).

Variables	Variables category	Frequency	Crude OR 95%CI	Adjusted OR 95% CI	P value of multivariate analysis
Sex of respondents	Female	204	1	1	
	Male	207	1.27(1.81–1.97)	1.19(0.78–4.23)	0.27
Travel History**	Yes	75	2.28(1.35,3.87)	2.59(1.24, 5.38)	0.001
	No	336	1	1	0
Ever heard about malaria	Yes	376	1	1	
	No	35	1.57(1.23–1.42)	1.24(0.65–4.58)	0.132
Had home visit by HEW	Yes	326	1	1	
	No	85	2.17(1.05–2.99)	1.17(0.45–3.35)	
Knowledge Score Level**	Poor	136	3.26(1.69,6.29)	5.09(2.26,11.50)	0.012
	Medium	198	0.77 (0.39,1.52)	0.89(0.38,2.07)	0.418
	Good	77	1	1	
Bed net ownership**	Yes	171	1	1	
	No	240	3.21(1.63,6.31)	4.67(2.11, 10.37)	0.003
Practice Score level**	Poor	241	1.91(1.19,3.06)	2.28(1.10,4.74)	0.004
	Good	170	1	1	
Family size	1–4	126	1	1	
	5–8	235	1.23(1.68-2-83)	1.15(0.43–3.97)	0.223
	≥8	50	1.14(1.01–1.41)	1.05(0.64–1.41)	0.153
Estimated distance from stagnant water(in meter)	<500	143	1.18 (0.71, 1.95)	1.87(0.93,3.76)	0.123
	500–1500	154	0.95 (0.44, 2.07)	3.32(1.13,9.76)	0.015
	1501–3000	43	0.51 (0.24, 1.064)	0.99(0.37,2.66)	0.132
	>3000	71	1	1	

** Has statistically significant association with slide positivity

## Discussion

The current study revealed that overall slide positivity of malaria in the study area among febrile cases was high (25.8%). This figure is much higher than the result of a study done in Maputo, Mozambique among febrile cases where 16% of the patients had a positive blood slide for malaria [23]. Regarding the types of *Plasmodium* species identified, *P*.*vivax* was dominant malaria species (71.7%) followed by *P*. *falciparum* which accounted for 25.5% of the cases. This finding is consistent with findings of studies in the high land fringes of Butajira, Jimma and Harar in Ethiopia where proportions of 62.5% 76%and 67.3% were reported respectively although malaria risk classification is different in Butajira [24, 25, 26]

Conversely, another study conducted in northern Ethiopia reported 75% of *P*. *falciparum* and 25% *P*.*vivax* cases were reported [11]. The reason why *P*.*vivax* dominated in the study area might be due to occurrence of relapses at different intervals as a result of the activation of liver-stage or if the febrile cases came from highly epidemic prone areas. Further research needs to be done on activation of hypnozoites for relapse in the population of study area. The finding might have implication as challenges to the malaria elimination strategy for three reasons. The first is that mosquitoes bite early in the evening, obtain blood meals outdoors and rest outdoors therefore ITNs and IRS may be less effective in reducing the transmission of *P*. *vivax* parasites. The second is that blood-stage infections of *P*. *vivax* often occur with low parasite densities and can be missed using routine microscopy or rapid diagnostic tests. The third is that primaquine requires a 14-day treatment course to which patients may not fully adhere. [2]

Concerning bed net ownership, 41.6% of the households had at least one bed net of any type. This finding is lower than the national malaria indicator survey where 55.2% households had at least one mosquito net (of any type) [11]. Similarly, a high percentage of ownership (89.1%) was reported by the study done in northern Ethiopia [27].

The majority of the respondents (80%) mentioned that sleeping under bed net prevents malaria. However, only 60.2% of them reported that they slept under bed nets in the previous night. This misconception might be due to a low frequency of home visit by the health extension workers [13]. This study also revealed that 39.8% of the individuals who owned a bed net reported that they were not using it appropriately. The reported reasons of the respondents for not using were insufficient supply (50.8%), insufficient number for family members (38.5%), exhaustion (7.7%) and the perception that there is no malaria in the area (3.1%). The reported reasons were among associated factors in another study done in Ethiopia [28].

Unexpectedly, only 26.5% of the respondents knew that infected *Anopheles* mosquito bite is the means for malaria transmission which is much lower than the findings of the studies elsewhere in Ethiopia [17, 29] and East African [18]. This low knowledge reported regarding infected *Anopheles* mosquito bite also contradicts other knowledge items of prevention and practice all of which suggest further intervention have to be done on raising the level of awareness. This might be due to lack of health education through effective communication means on malaria transmission methods prevention and control by health extension workers and other concerned bodies. Another possible reason could be due to respondents from highland areas (non-endemic areas) where malaria is not taken as a major health problem and hence they might not be well informed on its transmission means. Moreover, the historical decrease of malaria prevalence from the starting of roll back strategy in the country might result in lack of attention of knowledge and behavioral interventions.

Regarding self-reported treatment seeking behavior of the respondents, only 29.9% % had sought treatment within the recommended time. This finding is higher than the study done in Adami Tulu district in south-central Ethiopia [30]. However, this finding was extremely low from the expectation of higher home visit made by health extension workers as showed in descriptive finding of this study (see Table 2). This finding might suggest that more focus is needed for increasing treatment seeking behavior to support early detection and treatment of malaria cases while visiting homes.

Participants overall malaria knowledge and practice in this study is remarkably low i.e. 18.7% and 41.4%, respectively as compared to other findings in Ethiopia. The 2011 Malaria Indicator Survey reported that peoples’ knowledge and practice to malaria was 71.2% and 645% respectively [11, 16]. This finding is also low in the studies done in western and northern areas of Ethiopia [27, 29, 31]. The possible reason for the low knowledge and practice score in this study could be due to that the study area being malaria non-endemic and hence participants from highland localities might not have enough information about the disease and its prevention methods. Another implication is that health extension workers and the current community health development army might give less focus on malaria regarding health information communication strategy to fill gaps on knowledge and practice.

Among the factors analyzed in multivariate analysis, estimated distance of surface water away from residence was significantly associated with malaria positivity. This is consistent with the malaria studies conducted in Ethiopia [12, 29, 32,33]. Not using bed net (LLITNs) had also showed statistically significant association with malaria blood slide positivity which is similar with other studies in the country [11, 29, 32].

Poor level of practice of prevention and control measures of malaria showed a statistically significant association with the probability of getting malaria even though the scoring system was conservative. This is congruent with similar studies conducted indifferent region of Ethiopia [13]. Travel history also had a statistically significant association with blood slide positivity. This finding is consistent with the study done in central Ethiopia [34]. However the study is showed travel history associated with only *P*. *falciparum*.

## Conclusion

The present study revealed that malaria is still a major source of morbidity in the study area among febrile illnesses. Poor level of knowledge, poor prevention practices, not using bed net, travel history to endemic areas and residing near stagnant water were factors with associated malaria positivity in the study area. Therefore, implementers, policy makers and stakeholders should strengthen the services provided by community health development army, health extension service and health facilities services focusing on increasing malaria intervention coverage and mobilization of information, education and communication to increase knowledge about malaria transmission, prevention and control practices.
